# The Sunday Question

**Published:** 1875-01

**Authors:** 


### THE SUNDAY QUESTION.

Omniscience has ordained—and human observation has confirmed the wisdom of it—that the body does not get enough rest during the week, and the Sabbath has been appointed as compensatory.

Observant men who have had the control of large capital, or large numbers of workmen or cattle, have discovered in different nations and ages and in various occupations and callings, that more work is done in six days than in seven.

For a century business men insisted that bricks could not be made unless the fires were kept up for a week or more without intermission; it has of late been found that it can be done and to greater advantage. It has been left to within five years to demonstrate that it is more profitable to omit making cheese on Sunday than to keep it up continuously; that blast-furnaces make their owners rich sooner if Sunday is not employed in keeping up the fires.

The Third Avenue horse car company has found it to be a saving to the health and life of their animals to have substitutes for Sunday service. But how best to spend Sunday, morally and pysiologically, has not yet been satisfactorily shown, because certain general principles have not had their due weight. The Divine announcement was made in the beginning, "Thou shalt not do any work" on that consecrated day; the precept extending not only to the master but the servant and even the beasts of the field. Whatever is unnecessary work on Sunday is wrong.

Four thousand years later, the New Dispensation announced that "The Sabbath was made for man, and not man for the Sabbath;" that the day of rest—every seventh day—was set apart for the benefit of man, to promote his happiness and well-being. The upper classes, who are well-to-do, have no conception of the deeply joyous welcome with which all who work by the week or month or year greet the coming of Sunday. The rest intended to be secured by the institution of the Sabbath includes that of the body and mind and heart—the physical, mental, and moral nature of man.

The body is best rested by remaining in bed several hours longer on Sunday than on a week day; remaining in bed longer in the morning and retiring earlier at night. Instead of this an hour or two of sleep in the forenoon and a shorter nap in the afternoon is advantageous. If this is properly managed the preaching heard will do many times more good because the hearer will be more wide awake, the brain will be more active, the power of taking in ideas and of linking them together will be increased so that the sermon will be more easily comprehended and the entire service more enjoyable. Every one knows how impossible it is to enjoy a sermon when there is so much drowsiness that the eyes can scarcely be kept open. Our Sunday dinners, wrongly made the best of the week, too often send us to the afternoon sermon like gorged anacondas; attention is painful, appreciation impossible, and we come away unbenefited and uninstructed. But if we go to church after a good nap we listen with interest to the dullest sermon; the dullness, however, being nine times out of ten in the sleepy hearer, rather than in the minister. It is impossible for a man to be spiritual after a gluttonous meal.

A man will do more work in the long run by working ten consecutive hours a day than twenty, and will certainly do it better; so he can have more active and enjoyable devotional feeling during a few hours of religious service, if he has been fully rested in heart and brain, than in double the time, if half asleep. If persons would go to bed several hours sooner on Saturday night it would not be necessary to sleep on Sunday; but as the times are, it is not practicable for half the slaves in the United States, meaning thereby farmers' wives, who know very well, if men do not know it, that there is more work to do on Saturdays than during any other day in the week; so much more, in fact, that the necessity of working until eleven or twelve o'clock is oftener the rule than the exception ; and many, very many, city wives have the same experience.

SUNDAY DINNERS.—These should be the simplest and plainest of the week, thus allowing servants more time for rest ; giving over taxed stomachs more repose, to say nothing of the more perfect appreciation of the afternoon service ; but a clear head is a physiological impossibility to all those whose Sunday dinners are a feast of fat things, because they are tempted to eat so much that all the nervous energy of the system is compelled towards the stomach to help to digest the enormous load, leaving so little for supporting the brain operations, that the thinking faculties scarcely work at all ; hence the sleepiness after a hearty meal; and as we cannot alter nature's laws, we must adapt ourselves to the circumstances which surround us ; if we cannot quit work early on Saturday we had better sleep half of Sunday as a preparation for the highest religious enjoyment of the other half of the day.

SUNDAY RECREATIONS.—"Recreation" is, literally, a making over again, a second creation ; the word is usually applied to our minds, indicating a renewal or renovation of strength and activity, by a cessation from ordinary occupations which are in the nature of work, and engaging in something which is less of a task, and more of an enjoyment. But in the physiological sense of the word, Sunday excursions, pic-nics, going out to the Park, or a ride or walk into the country on the Sabbath day are not recreations, are not making it a day of rest. Any one must know, who has engaged in any of these, that he comes home, that all come home on Sunday night perfectly "fagged out," so tired, so weary, so dead, that he is glad to hurry off to bed, even more tired than after the close of an ordinary day's work, to say nothing of the money spent, which is sometimes more than a day's wages ; thus failing to get any of the extra rest of the Sabbath, but losing a day in life with the result that there is more exhaustion or Monday morning, than on any other day of the week; less alacrity, less disposition to go to work, instead of a feeling of vigor and energy, and a pent up power which makes its expenditure in labor a pleasure and a joy.

The very best way for the working-classes to spend Sunday is to stay at home and in the house, excepting so much time as may be required for attending public worship. Every unnecessary step taken is a loss, it is a waste of strength; and for the same reason all family visiting should be avoided, all feasting and parties, and if one must go out of the house, let it be to comfort or assist some one in sickness or trouble.

The actual in-door occupation of Sunday should be of a restful, passive character, such as would tend to rest of body, and composure of mind ; and these are best promoted by retiring to one's own room to read, and sleep, and lounge, and meditate, and sing. This would be rest and recreation—the recreation for which the Sabbath was clearly set apart.

SUNDAY SLAVERY.—To children and Sunday-School teachers the Sabbath is not a day of rest, but of toil, which in many cases makes it a burden. Between Sunday-School lessons and going to church, many children are altogether over-taxed in mind and body. Sunday-School at nine, church at eleven, and in the afternoon or at night, and the various preparations for these duties make Sunday a hard day instead of a day of rest, as it was ordained to be.

As things are, it would be more profitable for the over-worked of all classes to sleep half the day on Sunday and to spend the remainder in religious duties, for no human being can worship acceptably and enjoyably when half awake. A better method would be to inaugurate a plan of ceasing work at noon on Saturday so that the afternoon might be spent in special preparation for Sunday, so that all could go to bed shortly after sun-down, as many farmers do. A good beginning might be made by men of wealth and Christian principle taking the initiative by closing their places of business promptly at twelve o'clock on Saturday the year round, at the same time paying for the full week's work ; this would be a literal and a commendable "remembering" of the Sabbath day, by beginning a preparation for it, before it came.

SUNDAY READING.—It is always safe and restful to read appropriate selections from the Bible, and there are many other books which will tend to calm the mind and give peace and rest to the body and brain. We would gladly commend the religious newspapers, as superior Sunday literature, if we could do so honestly. As an editor we receive a good many of them in exchange, and have to admit that we find a good deal to condemn in most of them, along with much that is admirable. We find quack-medicines bolstered up, dubious life insurance concerns adroitly commended, and gambling "Bond" schemes given kindly prominence. We observe, with pain, constant and harsh denominational controversy, and personal editorial animosity, blended with discourteous and depreciative epithets. All this we deprecate of course, as do most readers, no doubt. But the matter contained in these religious exchanges of ours, is, in the main, too valuable to be wasted. So, after we have read the sheets, we send them into the kitchen for the benefit of the servants, to amuse, to entertain and to instruct. But our servants are Roman Catholics, against whom such harsh things are sometimes said in these sheets, that we either cut out the abuse or burn up the paper containing it, as no man or woman was ever induced to adopt another religion by being abused, ridiculed or knocked down ; besides, the meanest of all mean things is to insult the religious sentiment of those who wait upon us and are dependent on us. If we have any desire to win our servants to our religion we must invite them to it, we must conciliate them, win them over. It is not by any means a marvel that the Catholic girls, who come to this country from Ireland, adhere so heroically to the faith of their fathers, and through the bitterest of Winter weather seldom fail to attend the daylight Sabbath morning service ; and who shall say that they are not accepted ? What a mission might the religious newspaper perform, and what an efficient means of converting multitudes of those who serve in our homes, if conducted in a more conciliatory manner, more in the dignified spirit in which ministers of state, and national ambassadors conduct their correspondence, and arrange the differences which are constantly coming up between their separate countries! The religious press is now second only to the preaching of the Gospel, as a means of converting the world. The editor has a wider and a greater responsibility in proportion to the greater extent of his field. If the religious newspaper could be conducted without offense to our servants, who are of a different "Faith," and filled as they are, with so much that is good, it would be a means of keeping them at home on Sunday, of keeping them out of the streets, from Sunday walks, and Sunday visiting, and Sunday pic-nics. But if their feelings are hurt, if their religious sentiment is insulted, the very natural result is, that if the paper was given them to read, it would not only remain unopened, but would be regarded with a spirit of indignation. The whole subject of Sunday occupations and Sunday rest merits the serious consideration of thoughtful minds, for the glory of any nation is a well kept Sabbath, and where there is no Sabbath "there is no God."

